# Comparative Genomics of *Methanopyrus* sp. SNP6 and KOL6 Revealing Genomic Regions of Plasticity Implicated in Extremely Thermophilic Profiles

**DOI:** 10.3389/fmicb.2017.01278

**Published:** 2017-07-11

**Authors:** Zhiliang Yu, Yunting Ma, Weihong Zhong, Juanping Qiu, Jun Li

**Affiliations:** ^1^Department of Applied Biology, College of Biotechnology and Bioengineering, Zhejiang University of Technology Hangzhou, China; ^2^State Key Laboratory of Microbial Metabolism, Shanghai Jiao Tong University Shanghai, China

**Keywords:** *Methanopyrus*, genomic architecture, thermophilic adaption, environmental advantage, marine microbe

## Abstract

*Methanopyrus* spp. are usually isolated from harsh niches, such as high osmotic pressure and extreme temperature. However, the molecular mechanisms for their environmental adaption are poorly understood. Archaeal species is commonly considered as primitive organism. The evolutional placement of archaea is a fundamental and intriguing scientific question. We sequenced the genomes of *Methanopyrus* strains SNP6 and KOL6 isolated from the Atlantic and Iceland, respectively. Comparative genomic analysis revealed genetic diversity and instability implicated in niche adaption, including a number of transporter- and integrase/transposase-related genes. Pan-genome analysis also defined the gene pool of *Methanopyrus* spp., in addition of ~120-Kb genomic region of plasticity impacting cognate genomic architecture. We believe that *Methanopyrus* genomics could facilitate efficient investigation/recognition of archaeal phylogenetic diverse patterns, as well as improve understanding of biological roles and significance of these versatile microbes.

## Introduction

The family *Methanopyraceae* currently includes only one single genus with one single species, *Methanopyrus kandleri*, which was isolated from the sea floor locating at the base of a 2,000 m-deep “black smoker” chimney in the Gulf of California (Kurr et al., 1991). *Methanopyrus kandleri* is a rod-shaped chemolithoautotrophic methanogenic archaea which can grow at up to 110°C and even higher together with high hydrostatic pressure. Thus, *Methanopyrus kandleri* was considered as the most extreme thermophile among the methanogens (Oren, 2014). The cell wall of *M. kandleri* contains a rather unusual lipid, 2, 3-di-O-geranylgeranyl-*sn*-glycerol, speculating its primitiveness (Slesarev et al., 2002).

The phylogenetic position of *M. kandleri* has been disputed so much. Earlier studies have implied that *M. kandleri* holds more euryarchaeal signature features than any other known euryarchaeal rRNA in its 16S rRNA gene (Burggraf et al., 1991). Based on phylogeny from 16S rRNA, *M. kandleri* lineage was proposed to branch off proximal to the universal root of life, supporting that it is probably the Last Universal Common Ancestor (LUCA). However, other more comprehensive analysis cannot confirm its special evolutionary status of LUCA. According to the trees based on concatenated alignments of ribosomal proteins, as well as the trees from gene content, *M. kandleri* is consistently grouping with other methanogens (Slesarev et al., 2002). *Methanopyrus kandleri* shares the archive of genes involving methanogenesis and its operon associated with several methanogens, remarkably indicating that archaeal methanogens could be monophyletic (Slesarev et al., 2002). Moreover, close relationships of *M. kandleri* with the *Methanobacteriales* and the *Methanosarcinales* were thus proposed via gene order-based phylogeny (Luo et al., 2009).

Similar to most prokaryotic microorganisms, the genome of most methanogens usually consists of a single circular chromosome with sizes usually 1.6~5.7 Mb. In particular, the genomic sizes of *Methanobacteriales, Methanococcales*, and *Methanopyrales* are all around 1.7 Mb. The G+C content of methanogens is 30~65%, varying closely associated to their growth environment. In general, higher growth temperature is consistent with higher G+C content in archaeal genomics (Wang et al., 2014). Remarkably, the genomic size of the representative strain *M. kandleri* is smaller than other methanogens. Some regulatory and signaling systems presenting in other archaea are lack in *M. kandleri*, indicating that these systems are scaled down in *M. kandleri* (Oren, 2014). The proteins encoded by its genome have a high content of negatively charged amino acids, possibly adapting to high content of intracellular ions and helping the enzymes to maintain activity and stability. These embedded features probably influence the adaption capacities in extreme habitat.

Several *Methanopyrus* strains have been isolated from marine biotopes with high temperature. Our previous study focusing on the interparalog gene divergences of *Methanopyrus* isolates from different oceans suggested that isolates collected from Central Indian Ridge as the most primitive strain based on its genetic distance of gene fragment (Kurr et al., 1991; Yu et al., 2009). This study also revealed that there is a significant association between the genome architectures and the geographic characteristics. Although a number of studies showed that microbes display variable biogeographic patterns in the ocean (Agogue et al., 2011; Caporaso et al., 2011), little is understood about the geographic isolation impact on archaeal genome divergence.

In this report, we sequenced genomes of two *Methanopyrus* isolates (SNP6 and KOL6) and performed genomic comparison against *M. Kandleri* AV19 of which the genome had been previously published. This study is the first comparative genomic analysis for *Methanopyraceae* family, and aims to: (i) demonstrate the evolutionary divergence of *Methanopyrus* isolates with the whole-proteome sequence, and then (ii) illustrate the mechanism underlying their extreme environmental adaption capacities.

## Materials and methods

### Sources of strain and DNA isolation

*Methanopyrus* sp. SNP6 was isolated from the wall of active “black smokers” at the Mid Atlantic Ridge (“Snake Pit” site, 23.22°N; 44.57°W), and *Methanopyrus* sp. KOL6 was isolated from Kolbeinsey Ridge, Iceland (68.02°N; 17.08°W). Both are located in submarine hydrothermal areas (Penders, 2003). The source of *Methanopyrus* isolate samples and the method of DNA extraction were described in our previous study (Yu et al., 2009).

### Genome sequencing and assembly

*Methanopyrus* strains SNP6 and KOL6 were sequenced in this study on Illumina Mi-Seq system by using Mi-Seq Reagent Kit v3 (600 cycles). Paired-end reads of average 500-bp library length were assembled using SOAP*denovo* program (version 2.04) with given parameters (Figure S1). In the final assembly, contigs less than 25-bp in length were discarded. The sequencing coverages are 2635× for SNP6 and 1347× for KOL6. Sequence data from this study have been deposited at GenBank Whole Genome Shotgun (WGS) under accession numbers SAMN06246566 and SAMN06246254 for SNP6 and KOL6, respectively.

### Gene prediction and annotation

The open reading frames (ORFs) of *Methanopyrus* strains SNP6 and KOL6 were predicted by Glimmer 3.02 (Salzberg et al., 1998). All predicted ORFs were then annotated based on the NCBI Prokaryotic Genome Annotation Pipeline (Angiuoli et al., 2008) and Rapid Annotation using Subsystem Technology (RAST) (Aziz et al., 2008). The rRNA identification was performed with RNAmmer 1.2 software (Lagesen et al., 2007), and tRNAscan-SE (v1.21) was used to identify the tRNA genes (Lowe and Eddy, 1997).

### Core genome characterization and pan-genome analysis

All predicted protein sequences were grouped together and reciprocally compared using BLASTp program of NCBI-BLAST 2.4.0+ suite. The homologous protein pairs with *E*-value cut off 1 × e^−5^, percent of matching length ratio ≥30% and sequence identities ≥30% as previously described (Feng et al., 2015; Zheng et al., 2016) were used. The functional categories of identified proteins were inferred against the COG (Cluster of Orthologous Groups of proteins) databases (Tatusov et al., 1997).

### Sequence comparison

The genomes of selected strains were all-against-all compared using the Blast Ring Image Generator (BRIG) software (Alikhan et al., 2011). Regions with nucleotide sequence similarity above 50% are shown on the map. Four genomic islands (GIs) were identified based on comparative analysis in VRprofile (Li et al., 2017). The upstream and downstream regions of each GI were then retrieved and manually searched for the presence of conserved regions or signature genes (such as tRNA, integrase or transposase). Remarkably, although a draft genome (SNP6) with a number of contigs was involved in analysis, the completeness of the genome was more than 95% as a result of the high genome sequencing coverage. Meanwhile, all gene loss occurred inside contigs but not between contigs of SNP6.

### Phylogenetic tree construction

The phylogenetic tree based on whole-genome comparison was constructed by the CVTree online server. Component vectors of the Broussonetia papyrifera (CVTree) method is based on whole genome, without sequence alignment and phylogenetic relationships among species approaches. This program can quickly analyze the genetic relationship at the whole sequence level, and the classification status of the preliminary identification. Due to the rational application of the whole genome information, the CVTree method can be used to identify the relationship and classification of the species with high resolution.

## Results and discussion

### Genome sequencing and features

*Methanopyrus* sp. KOL6 genome was sequenced using Illumina Mi-Seq system with an Mi-Seq Reagent Kit (600 cycles), which resulted in 4,025,658 pair sequencing reads with an average length of 500-bp. After filtering, 98.60% of the total reads were assembled, resulting in one integrated scaffold of approximately 1.43 Mb.

Similarly, *Methanopyrus* sp. SNP6 genome was sequenced using Illumina Mi-Seq system, resulting in 7,474,310 pair sequencing reads with an average length of 400-bp. After filtering, 99.30% of the total reads were assembled, resulting in 9 contigs. Lastly, gaps between these contigs were closed by the Gapcloser program, generating two integrated scaffolds approximately 1.43-Mb and 8.8-Kb.

Given assembly strategies and quality control shown in Figure S1, the SNP6 and KOL6 genomes of sufficiently high quality have been acquired. Accordingly, a variety of assays were performed to obtain possible replication initial sites of SNP6 and KOL6 genomes, such as by using Ori-Finder 2 (an integrated prediction tool for replication origins in archaeal genomes; Luo et al., 2014) and DoriC (an updated database of bacterial and archaeal replication origins; Gao et al., 2013). Notably, the replication origin of SNP6 could not be characterized via Ori-Finder 2. Most probably, its replication origin is lost in the genomic gaps. In contrast, the replication origin of KOL6 could be identified by using Ori-Finder 2 results. However, significant hits of replication origins of both strains were both not found by using DoriC-embedded optional archaeal BLAST searches. These results emphasize one aspect of identification complexity/significance of archaeal replication origins (Gao et al., 2013; Luo et al., 2014).

Seven representative thermophilic *archaeon* strains were used to perform comparative genome analysis. These selected archaea are similar with *Methanopyrus* according to the phylogenetic trees based on 16S rRNA (Figure S2) and transfer RNA gene sequence (Xue et al., 2005; Table 1). Compared with other archaea, *Methanopyrus* sp. SNP6 and *Methanopyrus* sp. KOL6 are expectedly more similar with *M. kandleri* AV19. The genome sizes of both SNP6 and KOL6 are less than AV19, and they contain fewer predicted CDSs, as well as the decreased number of tRNA and rRNA. *Methanopyrus* spp. have higher GC content, as well as higher optimal growth temperature relative to other archaea. Most probably, the high GC content mediates the growth advantage in extreme thermophilic condition for the methanogens.

**Table 1:** Source, phenotype and genomic information for the nine thermophilic archaeon strains.

**Size (Mb)**	**GC (%)**	**CDSs**	**tRNA**	**rRNA**	OGT^*^	**Strain**
1.43	58.0	1,616	31	2	110°C	*Methanopyrus* sp. SNP6 (this study)
1.43	57.7	1,597	31	2	110°C	*Methanopyrus* sp. KOL6 (this study)
1.69	61.2	1,687	33	3	110°C	*Methanopyrus kandleri* AV19 Slesarev et al., 2002
1.74	31.4	1,699	37	6	85°C	*Methanococcus jannaschii* DSM 2661 Bult et al., 1996
1.75	49.5	1,746	38	7	65°C	*Methanothermobacter thermautotrophicus* Delta H Smith et al., 1997
1.74	41.9	1,802	46	4	98°C	*Pyrococcus horikoshii* OT3 Kawarabayasi et al., 1998
1.77	44.7	1,846	46	4	96°C	*Pyrococcus abyssi* GE5 Cohen et al., 2003
2.05	53.6	2,111	46	4	88°C	*Thermococcus gammatolerans* EJ3 Zivanovic et al., 2009
2.09	52.0	2,233	47	4	85°C	*Thermococcus kodakarensis* KOD1 Fukui et al., 2005

* *OGT, optimal growth temperature*.

### Genome comparison and phylogenetic analysis

**Figure 1:**
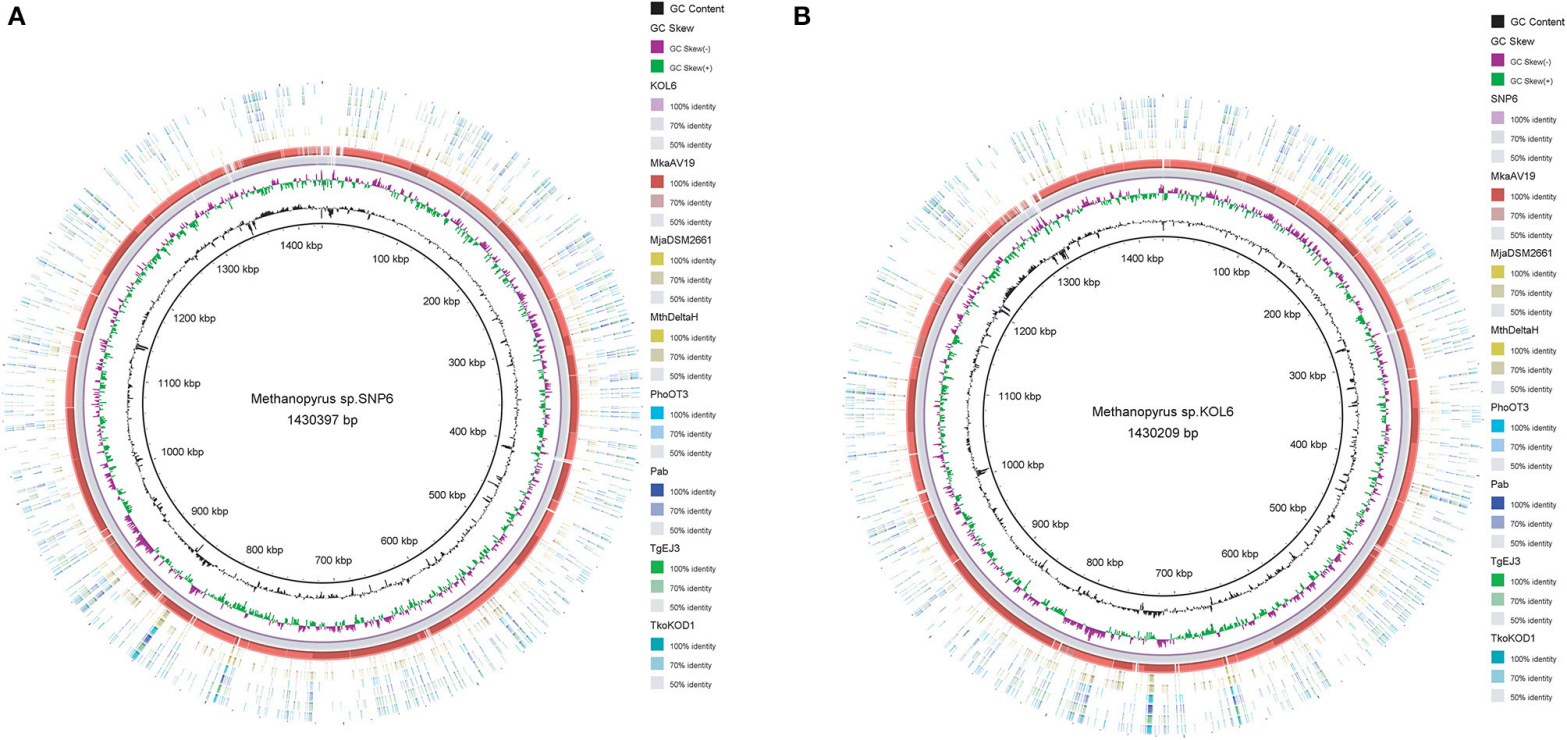
Circular graph of *Methanopyrus* sp. SNP6 **(A)** and KOL6 **(B)** compared with other eight archaeal genomes. From the inner to outer circles: (1) GC content plot with a gray circle representing 50%, (2) GC skew plot, (3) *Methanopyrus* sp. KOL6 **(A)**, (4) *Methanopyrus* sp. SNP6 **(B)**, (5) *Methanopyrus kandleri* AV19, (6) *Methanocaldococcus jannaschii* DSM 2661, (7) *Methanothermobacter thermautotrophicus*str. Delta H, (8) *Pyrococcus horikoshii*OT3, (9) *Pyrococcus abyssi*, (10) *Thermococcus gammatolerans* EJ3, (11) *Thermococcus kodakarensis* KOD1.

**Figure 2:**
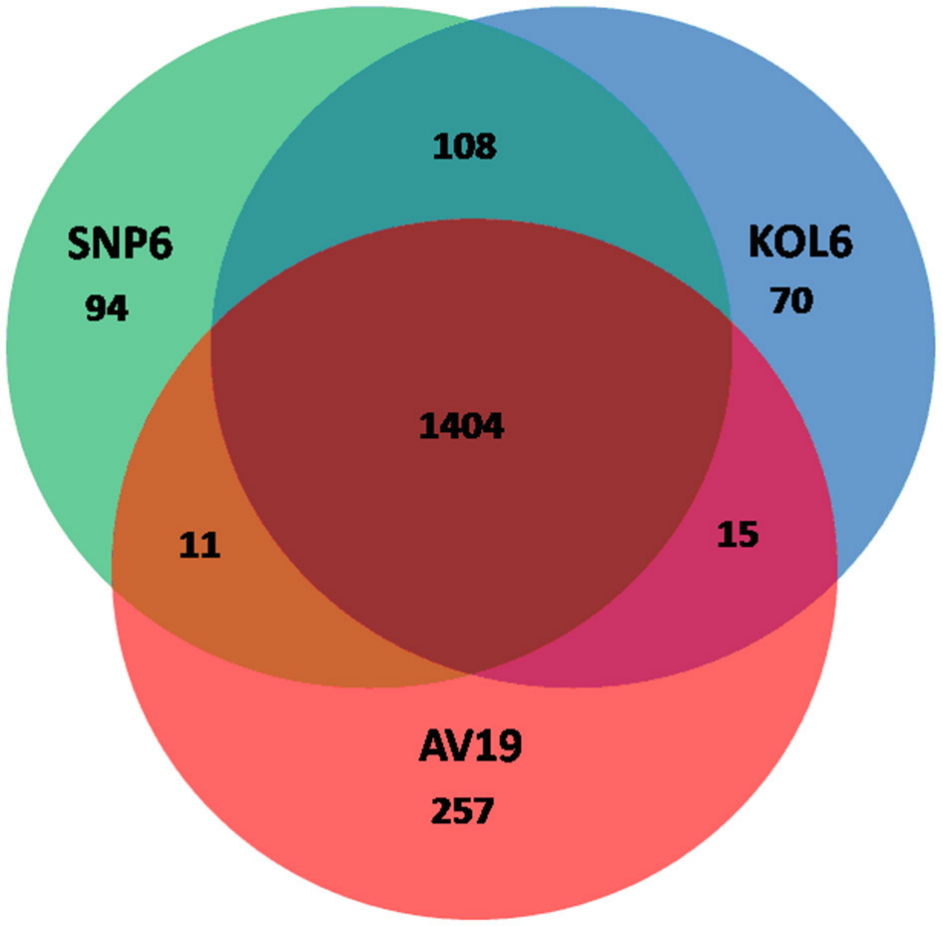
Venn plots presenting core and unique genes in each of *Methanopyrus kandleri* AV19, *Methanopyrus* strains SNP6 and KOL6.

Circular graphs were established to compare the genome sequence of the nine strains mentioned in Table 1. The sequence alignment indicated that SNP6 and KOL6 are significantly similar, and both have a high level of sequence conservation with *M. kandleri* AV19 (Figure 1). Based on the total set of genes from the three *Methanopyrus* isolates (SNP6, KOL6 and AV19), the pan-genome consists of a conserved core genome of 1,404 genes (Figure 2). The cumulative length of the core genome is approximately 1.14-Mb, covering >79% of each genome. In each genome, numbers of unique genes are from 70 to 257, accounting for 4.4% (KOL6), 5.8% (SNP6) and 15.2% (AV19), respectively, of its total gene numbers.

**Figure 3:**
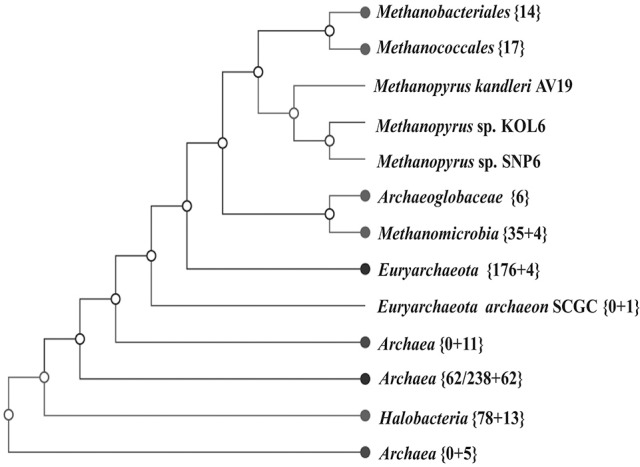
Unrooted neighbor-joining phylogenetic tree based on whole-genome protein sequence comparison via CVTree approaches, indicating SNP6 and KOL6 being closely-related strains of AV19.

A whole-genome tree based on gene-content comparison among 338 sequenced archaeal genomes archived in RefSeq was built by CVTree3 program (K=6). The result of preliminary classification showed that *Methanopyrus* strains SNP6 and KOL6 are closest to the hyperthermophilic archaeal methanogen *M. kandleri* AV19 and these three strains form an individual branch, which is much closer to *euryarchaeota* and consistently groups with other methanogens (Figure 3). Moreover, *Methanopyrus* isolates group with *Methanococcales* and *Methanobacteriales*, contradicting the early branching of *Methanopyrus* in the 16S rRNA tree (Figure S2). Phylogenetics based on whole-genome analysis revealed that the bacterial genomic architecture is highly susceptible to horizontal gene transfer (HGT) that has occurred between archaeal lineages (Philippe and Forterre, 1999). *Methanopyrus* groups with other thermophilic methanogens, presumably indicating the extensive HGT events across different lineages of methanogen from the same biotope (Brochier et al., 2004).

### Relationship of genomic architecture and habitat

**Figure 4:**
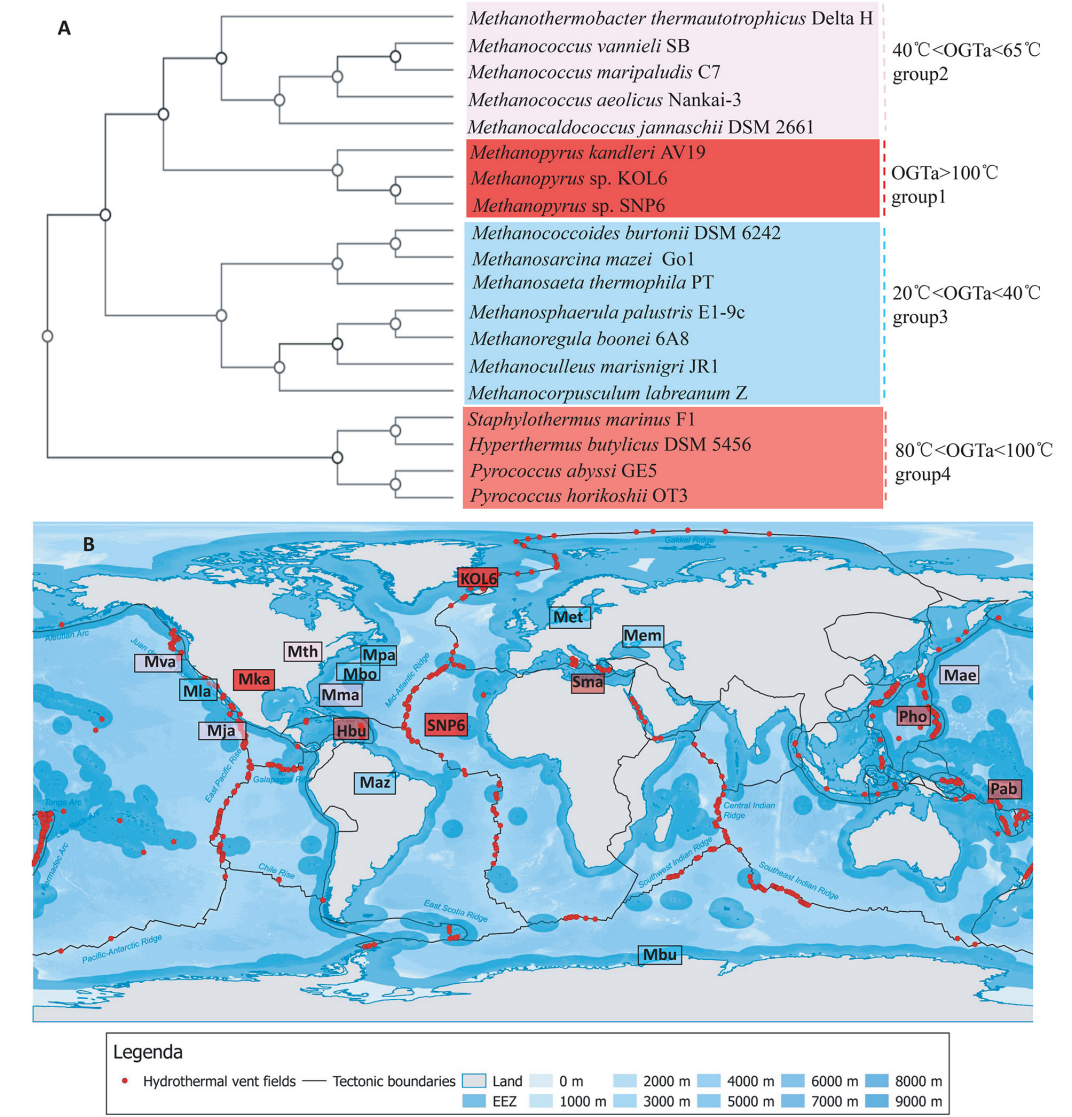
Whole-genome tree and regional distribution of the related strains. CVTree-generated whole-proteome level phylogenetics indicated 4 categories of closely-related genus **(A)**. Interestingly, group I-IV could respectively be in accordance with different habit niches (cold, hot, submarine volcano, or high osmotic pressure), implying genomic architecture influencing archaeal environmental advantages **(B)**. Species abbreviations: Archaea: Mth, *Methanothermobacter thermautotrophicus* Delta H (Smith et al., 1997); Mva, *Methanococcus vannieli* SB (Triscari and VanRaaphorst, 2010); Mma, *Methanococcus maripaludis* C7 (Leon and Larsen, 2011); Mae, *Methanococcus aeolicus* Nankai-3 (Copeland et al., 2007); Mja, *Methanococcus jannaschii* DSM 2661 (Bult et al., 1996); Mka, *Methanopyrus kandleri* AV19 (Slesarev et al., 2002); KOL6, *Methanopyrus* sp. KOL6; SNP6, *Methanopyrus* sp. SNP6; Mbu, *Methanococcoides burtonii* DSM 6242 (Franzmann et al., 1992); Maz, *Methanosarcina mazei* Go1 (Assis das Gracas et al., 2013); Met, *Methanosaeta thermophila* PT (Kato et al., 2014); Mpa, *Methanosphaerula palustris* E1-9c (Cadillo-Quiroz et al., 2009); Mbo, *Methanoregula boonei* 6A8 (Brauer et al., 2011); Mem, *Methanoculleus marisnigri* JR1 (Anderson et al., 2009); Mla, *Methanocorpusculum labreanum* Z (Anderson et al., 2009a); Sma, *Staphylothermus marinus* F1 (Anderson et al., 2009b); Hbu, *Hyperthermus butylicus* DSM 5456 (Brügger et al., 2007); Pab, *Pyrococcus abyssi* GE5 (Cohen et al., 2003); Pho, *Pyrococcus horikoshii* OT3. The source map of distribution of hydrothermal vent fields was created by DeDuijn. This map is under a CCBY-SA license and the URL link is https://creativecommons.org/licenses/by-sa/4.0/deed.en. **(B)** was generated by placing all the microorganisms at their isolated sites on the source map.

Several strains present close relationship with SNP6 and KOL6 according to Figure 3. Among them, we selected 19 representative strains whose source and phenotypic characteristics have been definitely characterized (Table S1), and then inferred a whole-genome level tree (Figure 4A). The tree consists of four groups and commonly existing niche features are shown, such as most of them are anaerobic (Table S1) and isolated from the Northern Hemisphere (Figure 4B). The strains pertaining to the same category are similar in growth temperature, such as group1 is higher than 100°C; group4 is between 80 and 100°C; group2 (except Mja) is between 40 and 65°C; group3 (except Met) is between 20 and 40°C. Furthermore, the group1 and group4 are all hyperthermophiles whose optimal growth temperatures are higher than 80°C, and all of them were isolated from submarine hydrothermal areas or volcanic fumaroles, implying the placement in evolution of hyperthermophiles. Moreover, the results revealed that group1 harbors a close relationship with group2 and group3, whose optimal growth temperatures (OGT) are lower (20 to 65°C) than group4. We found that these three subgroups share similar physiological properties, e.g., methane-producing and autotrophic. The group4 is all heterotrophy, and the gene cluster of methane metabolic pathway is also quite similar in group1 (Figure S3). We believe that genome architecture variation is closely related to microbiota environmental adaption, and there is a close relationship between genome similarity and geographic distance.

### Large genomic region of plasticity in SNP6 and KOL6

The whole genome protein sequences of SNP6 and KOL6 were aligned with AV19 by BLASTp program. There is a special genomic region containing a lot of unique genes (Figure 2, Figures S4, S5), about 80-Kb encoding 193 genes in SNP6 (Figure S5A, Table S2) and about 116-Kb coding for 163 genes in KOL6 (Figure S5B, Table S3). After re-annotation of the genes within this region, we found that only about 28.4% genes (in SNP6) and 56.4% (in KOL6) represent functional annotation, indicating that this region is relatively novel. Among these genes of the special genomic region, 114 genes in SNP6 are shared in AV19, but 80 genes in SNP6 are unique; 104 genes in KOL6 are shared in AV19, but 59 genes in KOL6 are unique. Most of these unique genes are related to inorganic ion transport, posttranslational modification or defense mechanisms, probably for adaption to extreme environments.

**Figure 5:**
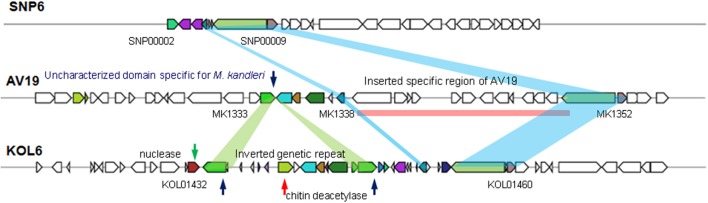
Genomic island GI4 homologs found in three *Methanopyrus* genomes via VRprofile.

HGT is common in bacteria, contributing to the genomic plasticity and possibly to environmental adaptation (Dobrindt et al., 2004). To better characterize genome plasticity and unique genome, four specific genomic regions larger than 10-Kb (except GI01 with 3.4-Kb) in size were identified. Especially, GI04 is absent or different in the corresponding regions of the two other genomes and designated here as the genomic region of plasticity (Figure 5).

Among these four GIs, GI04 contains more differences in the corresponding regions of the three strains than other GIs. In this GI, there are some partially conservative genes in the corresponding regions, though there exist significant differences on the whole, involving upstream and downstream regions. SNP6 and KOL6 lack a special region from gene MK1339 to MK1352 (Gene locus tag) compared to AV19. This special region contains 12 genes with unknown function and lacks in both SNP6 and KOL6 genome. We believe that these genes are not necessary for SNP6 and KOL6. Such genetic loss may reduce the metabolic burden. Notably, chitinase is a specific hydrolase for breaking down glycoside bonds on chitin (Kimyon et al., 2016), which is assigned as major composition on fungal cell wall. The nuclease encoding gene may mediate the horizontal transfer events within the focused region.

## Conclusions

In conclusion, *Methanopyrus* isolates SNP6 and KOL6 were sequenced, of which genomes are found to be closely related to representative *Methanopyrus* strain AV19. However, SNP6 and KOL6 each harbor a unique variable genomic region against AV19. Non-conserved genes within the focused regions are mainly metal ion transporter and/or horizontal transfer associated, proposed to influence adaption phenotypes. Regions of genomic instability in these two strains were further characterized via comparative analysis, since archaeal sequence features are not easily observed as previouly identified bacterial MGEs (mobile genetic events). Surprisingly, genomic architecture description revealed a close relationship between genetic diversity and archaeal habitat. Additionally, detailed genetic drift mechanism needs to be futher clarified, together with undefined function of unique genes in genomic regions of plasticity. Furthermore, the comparative analytical approaches in this study may enhance the sensitiveness of MGE definition in archaea, assisting idenification of genes with unknown function.

## Author contributions

ZY, JL, WZ, and JQ conceived and designed the study. ZY, YM, and JL performed the data collection and analysis. ZY, YM, JL, WZ, and JQ discussed, wrote and finalized the manuscript.

### Conflict of interest statement

The authors declare that the research was conducted in the absence of any commercial or financial relationships that could be construed as a potential conflict of interest.
