# Supplementary material for: Comparative Genomics of Methanopyrus sp. SNP6 and KOL6 Revealing Genomic Regions of Plasticity Implicated in Extremely Thermophilic Profiles
Source: Front Microbiol. 2017 Jul 11;8:1278. doi: 10.3389/fmicb.2017.01278 (PMC5504354; doi:10.3389/fmicb.2017.01278)
Supplement: Supplementary file 1 [file Table1.PDF]

Table S1. Source and phenotypic characteristics of the related strains.

Strain	**Origin**	**OGT***	**pH**	**Life style and other features**	**reference**
Methanothermobacter thermautotrophicus Delta H	Urbana, Illinois	65℃	_	Chemolito-autothroph, strict anaerobe, nitrogen-fixing methanogen	37
Methanococcus vannieli SB	San Francisco Bay	20-40℃	_	Anaerobic, methanogen,	23
Methanococcus maripaludis C7	South Carolina	35-40℃	6.5-8	Anaerobic, methanogen, autothrophic	24
Methanococcus aeolicus Nankai-3	Nankai Trough off the coast of Japan	42℃	7.2	Anaerobic, methanogen, autothrophic	25
Methanocaldococcus jannaschii DSM 2661	East Pacific Rise	85℃	5.2-7.0	Chemolito-autothrophic, strictly anaerobic, motile methanogen,	36
Methanopyrus kandleri AV19	Gulf of California<br>Sea floor at “black smoker” chimney	110℃	5.5-7 (6.5)	Chemolito-autothrophic, strictly anaerobic, methanogen, high intracellular salt concentration	3
Methanopyrus sp. KOL6	Kolbeinsey Ridge, Iceland<br>(submarine hydrothermal areas)	110℃	6.5-7.0	Chemolito-autothrophic, strictly anaerobic, methanogen	This study
Methanopyrus sp. SNP6	Mid Atlantic Ridge<br>(submarine hydrothermal areas)	110℃	6.5-7.0	Chemolito-autothrophic, strictly anaerobic, methanogen	This study
Methanococcoides burtonii DSM 6242	Ace Lake, Antarctica	23.3-29.5℃	7.7	Heterotrophy, methanogen	26
Methanosarcina mazei Go1	Amazonian Flooded Area	30℃	neutral	Methanogen, strictly anaerobic, autothrophic	27
Methanosaeta thermophila PT	Braunschweig,Germany	55℃	7.0	Methanogen, autothrophic, anaerobic	28
Methanosphaerula palustris E1-9c	New York State	28-30℃	5.5	Methanogen, autothrophic, anaerobic	29
Methanoregula boonei 6A8	New York State	35℃	5.1	Hydrogenotrophic methanogen, anaerobic,	30
Methanoculleus marisnigri JR1	Black Sea	20-25℃	6.0-7.5 (6.4)	Anaerobic，autothrophic, methanogen,	31
Methanocorpusculum labreanum Z	Los Angeles, California	37℃	6.5-7.5 (7.0)	Anaerobic, Chemolito-autothrophic, methanogen	32
Staphylothermus marinus F1	Vulcano Island, Italy<br>(geothermally heated sediments)	85℃	4.5-8.5 (6.5)	Heterotroph, anaerobic	33
Hyperthermus butylicus DSM 5456	Miguel in the Azores<br>(sea-bed with temperature up to112℃)	80-108℃	7.0	Anaerobic, heterotroph	34
Pyrococcus abyssi GE5	SW Pacific (North Fiji Basin)<br>(hydrothermal activity)	96℃	7.0	Anaerobic, motile heterotroph	35
Pyrococcus horikoshii OT3	Okinawa, Pacific Ocean<br>(hydrothermal vent)	98℃	7.0	Anaerobic, motile heterotroph	38

*****OGT: optimal growth temperature
