# Supplementary material for: Comparative Genomics of Methanopyrus sp. SNP6 and KOL6 Revealing Genomic Regions of Plasticity Implicated in Extremely Thermophilic Profiles
Source: Front Microbiol. 2017 Jul 11;8:1278. doi: 10.3389/fmicb.2017.01278 (PMC5504354; doi:10.3389/fmicb.2017.01278)
Supplement: Supplementary file 2 [file Table2.PDF]

Table S2. List of unique genes of SNP6 compared to strain AV19.

No	Synonym	Start	End	Strand	Length(aa)	Note
1	SNP00002	553	1341	+	262	_
2	SNP00003	1312	2091	-	259	_
3	SNP00004	2173	2898	-	241	_
4	SNP00005	2870	3220	-	116	hypothetical protein [*Methanopyrus kandleri*]
5	SNP00006	3326	3475	+	49	_
6	SNP00007	3495	3620	+	41	pleckstrin homology domain-containing family A member 1-like isoform X5/Cullin 1a
7	SNP00009	7249	7947	+	232	hypothetical protein [*Methanopyrus kandleri*]
8	SNP00010	8215	8787	+	190	_
9	SNP00011	8808	9515	+	235	_
10	SNP00012	9535	10167	+	210	component of a threonine efflux system [*Methanopyrus kandleri*]
11	SNP00013	10149	10322	-	57	_
12	SNP00041	37553	37888	-	111	hypothetical protein [*Methanopyrus kandleri]*
13	SNP00052	44739	45923	-	394	nicotinate phosphoribosyltransferase [*Methanopyrus kandleri*]
14	SNP00053	46045	46410	+	121	metal-binding protein [*Methanopyrus kandleri*]
15	SNP00054	46625	47383	-	252	hypothetical protein [*Methanopyrus kandleri*]
16	SNP00055	47380	48651	-	423	P-loop ATPase [*Methanopyrus* P-loop ATPase [*Methanopyrus kandleri* AV19]
17	SNP00095	90124	90240	+	38	
18	SNP00134	116918	117136	+	72	DNA-directed RNA polymerase subunit N [*Methanopyrus kandleri*]
19	SNP00139	121448	121951	-	167	_
20	SNP00141	123467	124141	-	224	flavoprotein [*Methanopyrus flavoprotein* [*Methanopyrus kandleri* AV19]
21	SNP00143	124599	125015	-	138	hypothetical protein [*Methanopyrus kandleri*]
22	SNP00144	125169	125423	-	84	_
23	SNP00146	126925	127848	+	307	type I restriction endonuclease EcoEI subunit S [*Methanopyrus kandleri*]
24	SNP00147	127861	128559	+	232	nitroreductase [*Methanopyrus* nitroreductase [*Methanopyrus kandleri* AV19]
25	SNP00148	128648	129238	+	196	rRNA/ tRNA methylase [*Methanopyrus kandler*i]
26	SNP00149	129294	129602	+	102	dihydroorotate dehydrogenase [*Methanopyrus kandleri*]
27	SNP00150	129615	130979	-	454	hypothetical protein [*Methanopyrus kandleri*]
28	SNP00155	134262	134453	-	63	_
29	SNP00187	155691	156611	+	306	formylmethanofuran dehydrogenase FwuB [*Methanopyrus kandleri*]
30	SNP00188	156727	157038	+	103	Zn-ribbon-containing protein [*Methanopyrus kandleri*]

31	SNP00221	192715	192870	-	51	thioredoxin reductase [*Methanopyrus kandleri*]
32	SNP00224	194615	194833	+	72	_
33	SNP00227	197890	198354	+	154	deoxyuridine 5'-triphosphate nucleotidohydrolase [*Methanopyrus kandleri*]
34	SNP00229	199132	199269	-	45	_
35	SNP00278	238807	238962	-	51	50S ribosomal protein L39 [*Methanopyrus kandleri*]
36	SNP00288	245260	246693	+	477	hypothetical protein [*Methanopyrus kandleri*]
37	SNP00291	248854	249042	+	62	_
38	SNP00295	252103	252375	-	90	_
39	SNP00302	259300	259470	-	56	ferredoxin [*Phascolarctobacterium* sp. CAG:266]
40	SNP00304	260893	261039	+	48	_
41	SNP00306	262986	263132	-	48	hypothetical protein [*Methanopyrus kandleri*]
42	SNP00307	263142	263348	-	68	_
43	SNP00328	283732	284001	+	89	hypothetical protein [*Methanopyrus kandleri*]
44	SNP00453	397852	398268	+	138	Holliday junction resolvase, type [*Methanopyrus kandleri*]
45	SNP00455	399886	400425	+	179	hypothetical protein [*Methanopyrus kandleri*]
46	SNP00456	400412	401842	+	476	hypothetical protein [*Methanopyrus kandleri*]
47	SNP00497	447936	448055	-	39	_
48	SNP00498	448039	448179	-	46	_
49	SNP00512	464444	465073	-	209	_
50	SNP00513	465077	465205	+	42	hypothetical protein [*Methanopyrus kandleri*]
51	SNP00526	474222	474482	-	86	metal-dependent hydrolase [*Methanopyrus kandleri*]
52	SNP00527	474639	474752	+	37	_
53	SNP00532	477510	477647	-	45	_
54	SNP00539	483497	483667	+	56	_
55	SNP00540	483802	483918	+	38	_
56	SNP00541	483872	484018	+	48	_
57	SNP00563	504822	504935	-	37	_
58	SNP00586	521948	522571	+	207	RNA methylase [*Methanopyrus* RNA methylase [*Methanopyrus kandleri* AV19]
59	SNP00593	528793	530565	-	590	hypothetical protein [*Methanopyrus kandleri*]
60	SNP00598	535201	535434	-	77	_
61	SNP00607	546009	546716	-	235	hypothetical protein [*Methanopyrus kandleri*]
62	SNP00615	553188	554111	-	307	formylmethanofuran dehydrogenase FwuB [*Methanopyrus kandleri*]
63	SNP00618	555425	555538	-	37	_

64	SNP00630	562902	563393	+	163	Predicted membrane protein
65	SNP00640	571796	573820	+	674	hypothetical protein [*Methanopyrus kandleri*]
66	SNP00653	584171	584374	+	67	_
67	SNP00678	606365	606508	+	47	_
68	SNP00679	606764	607594	+	276	chromosome partitioning ATPase [*Methanopyrus kandleri*]
69	SNP00680	607594	608319	+	241	hypothetical protein [*Methanopyrus kandleri*]
70	SNP00682	610128	610334	+	68	hypothetical protein [*Methanopyrus kandleri*]
71	SNP00698	624481	624666	-	61	_
72	SNP00701	627905	628267	+	120	_
73	SNP00705	630195	630386	+	63	_
74	SNP00708	633279	633431	+	50	hypothetical protein [*Methanopyrus kandleri*]
75	SNP00713	634862	635017	-	51	DNA-directed RNA polymerase subunit P [*Methanopyrus kandleri*]
76	SNP00746	658712	658858	+	48	Uncharacterized membrane protein/domain
77	SNP00749	659820	660113	+	97	hypothetical protein [*Methanopyrus kandleri*]
78	SNP00769	673651	673914	-	87	hypothetical protein [*Methanopyrus kandleri]*
79	SNP00776	678677	679417	-	246	hypothetical protein [*Methanopyrus kandleri*]
80	SNP00801	698631	699071	-	146	hypothetical protein [*Methanopyrus kandleri*]
81	SNP00883	771605	772330	+	241	coenzyme F420-reducing hydrogenase, gamma subunit [*Methanopyrus kandleri*]
82	SNP00885	773269	773733	+	154	hypothetical protein [Methanopyrus kandleri]
83	SNP00886	774042	774170	+	42	_
84	SNP00897	782510	783502	+	330	cation transporter [*Methanobacterium lacus*]
85	SNP00899	786481	786663	+	60	_
86	SNP00900	786682	786804	+	40	_
87	SNP00901	786779	786922	-	47	_
88	SNP00917	800964	801155	+	63	_
89	SNP00921	803756	803872	+	38	_
90	SNP00922	803880	804587	-	235	hypothetical protein [*Methanopyrus kandleri*]
91	SNP00937	816841	817452	+	203	hypothetical protein [*Methanopyrus kandleri*]
92	SNP00948	826815	826958	-	47	_transposase
93	SNP00949	827100	827234	+	44	_
94	SNP00953	830159	830326	-	55	Uncharacterized membrane protein specific for *M.kandleri*, MK-31 family
95	SNP00954	830504	830617	-	37	_
96	SNP00982	858998	859117	+	39	_

97	SNP00983	859139	859252	-	37	Predicted metabolic regulator containing two V4R domains
98	SNP00992	865064	866452	+	462	_
99	SNP00994	867300	868409	+	369	hypothetical protein [*Methanopyrus kandleri*]
100	SNP01024	895213	895371	+	52	tRNA/rRNA cytosine-C5-methylase [*Methanopyrus kandleri*]
101	SNP01025	895491	895661	+	56	_
102	SNP01036	904649	904810	+	53	_
103	SNP01052	927195	927344	-	49	hypothetical protein [*Methanopyrus kandleri*]
104	SNP01053	927380	927574	-	64	hypothetical protein [*Methanopyrus kandleri*]
105	SNP01054	927559	928530	-	323	prepilin-cleaving (type IV) signal peptidase [*Methanopyrus kandleri*]
106	SNP01056	929582	929830	-	82	_
107	SNP01057	929980	930207	-	75	Predicted secreted protein specific for *M.kandleri*
108	SNP01058	930270	930458	-	62	_
109	SNP01059	930459	931451	-	330	hypothetical protein [*Methanopyrus kandleri*]
110	SNP01060	931464	932735	-	423	hypothetical protein [*Methanopyrus kandleri*]
111	SNP01063	936790	938031	-	413	_
112	SNP01069	943293	945878	-	861	hypothetical protein [*Methanopyrus kandleri*]
113	SNP01191	1059448	1060725	-	425	hypothetical protein [*Methanopyrus kandleri*]
114	SNP01201	1066249	1066368	-	39	membrane protease subunit stomatin/prohibitin-like protein [*Methanopyrus kandleri* AV19]
115	SNP01240	1097102	1097398	+	98	transcriptional regulator [*Methanopyrus kandleri*]
116	SNP01249	1104913	1105716	+	267	DNA-modification methylase, partial [*Methanopyrus kandleri*]
117	SNP01250	1105997	1107394	-	465	Fe-S oxidoreductase [*Methanopyrus kandleri*]
118	SNP01257	1119669	1120097	+	142	ferredoxin [*Methanopyrus kandleri* AV19]
119	SNP01258	1120116	1120232	-	38	_
120	SNP01259	1120277	1120453	+	58	_
121	SNP01265	1128538	1128765	+	75	hypothetical protein [*Methanopyrus kandleri*]
122	SNP01266	1128923	1129066	+	47	_
123	SNP01270	1134798	1135226	-	142	_
124	SNP01276	1141104	1141283	-	59	_
125	SNP01286	1152001	1152135	-	44	prepilin-type N-terminal cleavage/methylation domain-containing protein
126	SNP01297	1160095	1160250	-	51	_
127	SNP01298	1160318	1160527	+	69	histidine kinase
128	SNP01303	1164897	1165103	-	68	_
129	SNP01304	1165221	1165388	+	55	_

130	SNP01349	1205360	1205527	+	55	_
131	SNP01377	1233194	1233376	-	60	hypothetical protein [*Methanopyrus kandleri*]
132	SNP01387	1239867	1240046	+	59	_
133	SNP01412	1272442	1272615	+	57	damage-inducible protein
134	SNP01416	1276808	1277107	-	99	hypothetical protein [*Methanopyrus kandleri*]
135	SNP01417	1277268	1277840	+	190	nucleotide kinase related to CMP and AMP kinase [*Methanopyrus kandleri* AV19]
136	SNP01424	1282126	1282239	+	37	_
137	SNP01434	1291524	1291700	+	58	_
138	SNP01437	1293734	1293889	-	51	4-vinyl reductase
139	SNP01441	1295104	1295553	+	149	_
140	SNP01453	1308393	1308512	-	39	_
141	SNP01454	1308511	1308645	+	44	_
142	SNP01455	1308679	1308828	+	49	_
143	SNP01461	1316196	1316492	-	98	_
144	SNP01462	1316550	1316738	-	62	hypothetical protein [*Methanopyrus kandleri*]
145	SNP01464	1318150	1318299	-	49	_
146	SNP01465	1318449	1318562	+	37	_
147	SNP01468	1320730	1321032	+	100	hypothetical protein [*Methanopyrus kandleri*]
148	SNP01469	1321265	1321507	-	80	transcription regulator [*Methanopyrus kandleri*]
149	SNP01480	1330625	1331200	-	191	hypothetical protein [*Methanopyrus kandleri*]
150	SNP01481	1331536	1331694	-	52	_
151	SNP01482	1331676	1333709	-	677	hypothetical protein [*Methanopyrus kandleri]*
152	SNP01483	1333678	1333878	-	66	hypothetical protein [*Methanopyrus kandleri*]
153	SNP01485	1335421	1335543	+	40	_
154	SNP01486	1336147	1336857	+	236	protease or amidase [*Methanopyrus kandleri*]
155	SNP01487	1336981	1337157	-	58	_
156	SNP01488	1337154	1337369	-	71	_
157	SNP01489	1338322	1338435	-	37	_
158	SNP01490	1338716	1338877	-	53	Predicted membrane protein specific for *M.kandleri*, MK-13 family, a frameshift
159	SNP01491	1339810	1341198	-	462	Fe-S oxidoreductase [*Methanopyrus kandleri]*
160	SNP01495	1345218	1345334	-	38	_
161	SNP01496	1345503	1345700	-	65	DNA polymerase beta family nucleotidyltransferase [*Methanopyrus kandleri*]
162	SNP01498	1345927	1346148	+	73	_

163	SNP01499	1346170	1346307	-	45	_
164	SNP01500	1346661	1346789	+	42	_
165	SNP01501	1346986	1347108	+	40	_
166	SNP01502	1347125	1347349	-	74	_
167	SNP01503	1347495	1347668	-	57	_
168	SNP01504	1347719	1348246	+	175	hypothetical protein [*Methanopyrus kandleri*]
169	SNP01505	1348318	1348515	-	65	rubrerythrin, partial [*Methanopyrus kandleri*]
170	SNP01549	1382260	1382733	+	157	hypothetical protein [*Methanopyrus kandleri*]
171	SNP01551	1383310	1383726	+	138	hypothetical protein [*Methanopyrus kandleri*]
172	SNP01553	1384626	1384742	+	38	_
173	SNP01555	1386564	1386698	+	44	preprotein translocase subunit TatC
174	SNP01570	1397679	1397810	+	43	membrane protein
175	SNP01571	1397956	1398090	+	44	_
176	SNP01574	1399923	1400204	+	93	_
177	SNP01575	1400383	1400496	+	37	_
178	SNP01576	1400606	1400803	+	65	_
179	SNP01577	1400847	1401083	+	78	type I restriction modification DNA specificity domain protein
180	SNP01578	1401215	1401559	+	114	hypothetical protein [*Methanopyrus kandleri*]
181	SNP01579	1401605	1401907	+	100	hypothetical protein [*Methanopyrus kandleri*]
182	SNP01581	1402800	1402937	-	45	_
183	SNP01583	1404305	1404460	+	51	_
184	SNP01585	1405078	1406241	-	387	coenzyme F420-reducing hydrogenase, beta subunit [*Methanopyrus kandleri*]
185	SNP01588	1408899	1409051	+	50	Glutathione peroxidase 1
186	SNP01589	1409757	1409885	-	42	_
187	SNP01592	1413049	1413309	-	86	_
188	SNP01595	1414973	1415140	+	55	_
189	SNP01598	1417336	1417479	+	47	hypothetical protein [*Methanopyrus kandleri*]
190	SNP01599	1417571	1418569	+	332	hypothetical protein [*Methanopyrus kandleri*]
191	SNP01600	1418864	1419130	-	88	transcriptional regulator
192	SNP01602	1420155	1420958	-	267	_
193	SNP01603	1420973	1421089	-	38	_
