# Supplementary material for: Comparative Genomics of Methanopyrus sp. SNP6 and KOL6 Revealing Genomic Regions of Plasticity Implicated in Extremely Thermophilic Profiles
Source: Front Microbiol. 2017 Jul 11;8:1278. doi: 10.3389/fmicb.2017.01278 (PMC5504354; doi:10.3389/fmicb.2017.01278)
Supplement: Supplementary file 3 [file Table3.PDF]

Table S3. List of unique genes of KOL6 compared to strain AV19.

No	Synonym	Start	End	Strand	Length (aa)	Note
1	KOL01295	1180748	1180867	-	39	_
2	KOL01296	1180866	1181000	+	44	CBS-domain-containing protein [*Methanopyrus kandleri* AV19]
3	KOL01297	1181034	1181183	+	49	CBS-domain-containing protein [*Methanopyrus kandleri* AV19]
4	KOL01298	1181227	1183269	+	680	N-methylhydantoinase A [*Methanopyrus kandleri*]
5	KOL01299	1183804	1185261	-	485	N-methylhydantoinase A [*Methanopyrus kandleri*]
6	KOL01300	1185331	1185756	-	141	Uncharacterized domain specific for *M.kandleri*, MK_11 [*Methanopyrus kandleri* AV19]
7	KOL01301	1186984	1188111	-	375	Uncharacterized protein specific for *M.kandleri*, MK-7 family [*Methanopyrus kandleri* AV19]
8	KOL01302	1188184	1188327	-	47	_
9	KOL01303	1188324	1188464	-	46	_
10	KOL01304	1188576	1188833	-	85	_
11	KOL01305	1188841	1189083	-	80	Uncharacterized protein specific for *M.kandleri*, MK-35 family [*Methanopyrus kandleri* AV19]
12	KOL01306	1189346	1190479	-	377	Uncharacterized protein specific for *M.kandleri*, MK-7 family [*Methanopyrus kandleri* AV19]
13	KOL01307	1190666	1190785	+	39	_
14	KOL01308	1190782	1190895	+	37	_
15	KOL01309	1190907	1191944	+	345	Predicted extracellular polysaccharide hydrolase of the endo alpha-1,4 polygalactosaminidase family
16	KOL01310	1191930	1192076	-	48	_
17	KOL01311	1192126	1193364	+	412	Uncharacterized protein specific for *M.kandleri*, MK-32 family [*Methanopyrus kandleri* AV19]
18	KOL01312	1193595	1193948	-	117	Predicted transcriptional regulator containing a HTH DNA-binding domain [*Methanopyrus kandleri* AV19]
19	KOL01313	1194349	1195266	+	305	Predicted ATPase of the PP-loop superfamily implicated in cell cycle control [*Methanopyrus kandleri* AV19]
20	KOL01314	1195258	1195998	-	246	Uncharacterized membrane protein specific for *M.kandleri*, MK-4 family [*Methanopyrus kandleri* AV19]
21	KOL01315	1196002	1197189	-	395	RecName: Full=UPF0272 protein MK1105
22	KOL01316	1197262	1197588	+	108	Nitrogen regulatory protein PII homolog [*Methanopyrus kandleri* AV19]
23	KOL01317	1197585	1198556	-	323	Predicted metabolic regulator containing two V4R domains [*Methanopyrus kandleri* AV19]
24	KOL01318	1198734	1200149	-	471	NAD-dependent aldehyde dehydrogenase [*Methanopyrus kandleri* AV19]
25	KOL01319	1200183	1200962	-	259	Uncharacterized protein MK1109 [*Methanopyrus kandleri* AV19]
26	KOL01320	1201340	1202287	-	315	Uncharacterized protein specific for *M.kandleri*, MK-8 family [*Methanopyrus kandleri* AV19]
27	KOL01321	1203033	1203608	-	191	Uncharacterized protein specific for *M.kandleri*, MK-22 family [*Methanopyrus kandleri* AV19]
28	KOL01322	1203955	1204125	-	56	_
29	KOL01323	1205749	1206834	-	361	Permease of the major facilitator superfamily [*Methanopyrus kandleri* AV19]
30	KOL01324	1207669	1208238	+	189	Predicted protease or amidase [*Methanopyrus kandleri* AV19]

31	KOL01325	1208241	1208360	+	39	Predicted protease or amidase [*Methanopyrus kandleri* AV19]
32	KOL01326	1208485	1208874	-	129	_
33	KOL01327	1209826	1210089	-	87	Predicted membrane protein specific for *M.kandleri*, MK-13 family, a frameshift [*Methanopyrus kandleri* AV19]
34	KOL01328	1211308	1212696	-	462	Fe-S oxidoreductase [*Methanopyrus kandleri* AV19]
35	KOL01329	1213176	1214648	+	490	Predicted glycosyltransferase [*Methanopyrus kandleri* AV19]
36	KOL01330	1214655	1216172	+	505	Uncharacterized membrane protein specific for *M.kandleri*, MK-16 family [*Methanopyrus kandleri* AV19]
37	KOL01331	1216736	1216852	-	38	_
38	KOL01332	1217021	1217218	-	65	Predicted nucleotidyltransferase of the DNA polymerase beta family [*Methanopyrus kandleri* AV19]
39	KOL01333	1217230	1217466	-	78	_
40	KOL01334	1217681	1217803	-	40	_
41	KOL01335	1218066	1218194	+	42	_
42	KOL01336	1218236	1218385	+	49	_
43	KOL01337	1218399	1218512	+	37	_
44	KOL01338	1218650	1218787	-	45	_
45	KOL01339	1218901	1219074	-	57	_
46	KOL01340	1219125	1219652	+	175	Uncharacterized membrane protein [*Methanopyrus kandleri* AV19]
47	KOL01341	1219947	1220396	+	149	Rubrerythrin [*Methanopyrus kandleri* AV19]
48	KOL01342	1220923	1222875	-	650	Ferrous ion uptake system subunit, predicted GTPase [*Methanopyrus kandleri* AV19]
49	KOL01343	1222880	1223107	-	75	ferrous ion uptake system subunit [*Methanopyrus kandleri*]
50	KOL01344	1223152	1224492	-	446	Acyl-CoA synthetase (NDP forming) [*Methanopyrus kandleri* AV19]
51	KOL01345	1224497	1224868	-	123	CBS domain-containing protein [*Methanopyrus kandleri*]
52	KOL01346	1224949	1225353	+	134	CBS-domain [*Methanopyrus kandleri* AV19]
53	KOL01347	1225399	1225575	+	58	ferredoxin [*Methanopyrus* ferredoxin [*Methanopyrus kandleri* AV19]
54	KOL01348	1225582	1225821	+	79	Zn-ribbon-containing protein [*Methanopyrus kandleri*]
55	KOL01349	1225822	1226151	+	109	Uncharacterized conserved protein [*Methanopyrus kandleri* AV19]
56	KOL01350	1226159	1226947	+	262	MinD superfamily P-loop ATPase containing an inserted ferredoxin domain [*Methanopyrus kandleri* AV19]
57	KOL01351	1226963	1227751	+	262	MinD superfamily P-loop ATPase containing an inserted ferredoxin domain [*Methanopyrus kandleri* AV19]
58	KOL01352	1227797	1228516	+	239	Uncharacterized protein MK1238 [*Methanopyrus kandleri* AV19]
59	KOL01353	1228520	1229200	+	226	RecName: Full=UPF0278 protein MK1237
60	KOL01354	1229197	1230633	+	478	Acetolactate synthase large subunit homolog [*Methanopyrus kandleri* AV19]
61	KOL01355	1230747	1231562	+	271	Predicted endonuclease of the RecB family [*Methanopyrus kandler*i AV19]
62	KOL01356	1231538	1232572	-	344	Uncharacterized protein MK1234 [*Methanopyrus kandleri* AV19]
63	KOL01357	1232720	1233508	+	262	ATPase subunit of an iron-regulated ABC-type transporter [*Methanopyrus kandleri* AV19]

64	KOL01358	1233493	1234416	+	307	Membrane subunit of an iron-regulated ABC-type transporter [*Methanopyrus kandleri* AV19]
65	KOL01359	1234422	1235081	+	219	FKBP-type peptidyl-prolyl cis-trans isomerase [*Methanopyrus kandleri* AV19]
66	KOL01360	1235071	1236132	-	353	RecName: Full=Glycerol-1-phosphate dehydrogenase [NAD(P)+]; Short=G1P dehydrogenase; Short=G1PDH; AltName: Full=Enantiomeric glycerophosphate synthase; AltName: Full=sn-glycerol-1-phosphate dehydrogenase
67	KOL01361	1236162	1237565	+	467	RecName: Full=Proline--tRNA ligase; AltName: Full=Prolyl-tRNA synthetase; Short=ProRS
68	KOL01362	1237591	1238241	+	216	RecName: Full=Proteasome subunit beta; AltName: Full=20S proteasome beta subunit; AltName: Full=Proteasome core protein PsmB; Flags: Precursor
69	KOL01363	1238305	1240263	+	652	Predicted metal-dependent RNase, consists of a metallo-beta-lactamase domain and an RNA-binding KH domain *[Methanopyrus*
70	KOL01364	1240277	1241317	+	346	RecName: Full=Phosphoribosylformylglycinamidine cyclo-ligase; AltName: Full=AIR synthase; AltName: Full=AIRS; AltName: Full=Phosphoribosyl-aminoimidazole synthetase
71	KOL01365	1241328	1241723	-	131	RecName: Full=50S ribosomal protein L32e
72	KOL01366	1241736	1242350	-	204	RecName: Full=30S ribosomal protein S8
73	KOL01367	1242364	1242756	-	130	30S ribosomal protein S8 [*Methanopyrus kandleri]*
74	KOL01368	1242772	1242918	-	48	30S ribosomal protein S14 [*Methanopyrus kandleri*]
75	KOL01369	1242933	1243493	-	186	50S ribosomal protein L5 [*Methanopyrus kandleri*]
76	KOL01370	1243505	1244287	-	260	30S ribosomal protein S4 [*Methanopyrus kandleri*]
77	KOL01371	1244302	1244748	-	148	50S ribosomal protein L24 [*Methanopyrus kandleri]*
78	KOL01372	1244762	1245163	-	133	50S ribosomal protein L14 [*Methanopyrus kandleri*]
79	KOL01373	1245183	1245515	-	110	30S ribosomal protein S17 [*Methanopyrus kandleri*]
80	KOL01374	1245530	1245853	-	107	RecName: Full=Ribonuclease P protein component 1; Short=RNase P component 1; AltName: Full=Rpp29
81	KOL01375	1245853	1246284	+	143	_
82	KOL01376	1246274	1247353	-	359	Isopropylmalate dehydrogenase [*Methanopyrus kandleri* AV19]
83	KOL01377	1247350	1248702	-	450	RecName: Full=UPF0210 protein MK1214
84	KOL01378	1248708	1248980	-	90	RecName: Full=UPF0237 protein MK1213
85	KOL01379	1249205	1250014	+	269	RecName: Full=Undecaprenyl-diphosphatase; AltName: Full=Undecaprenyl pyrophosphate phosphatase
86	KOL01380	1250020	1250412	+	130	RecName: Full=Putative fluoride ion transporter CrcB
87	KOL01381	1250409	1250747	+	112	Uncharacterized conserved protein [*Methanopyrus kandleri* AV19]
88	KOL01382	1250752	1251945	+	397	RecName: Full=Putative homocitrate synthase AksA; AltName: Full=(R)-homo(2)citrate synthase; AltName: Full=(R)-homo(3)citrate synthase
89	KOL01383	1252462	1253670	+	402	3-isopropylmalate dehydratase large subunit [*Methanopyrus kandleri*]
90	KOL01384	1253676	1254149	+	157	Predicted membrane protein [*Methanopyrus kandleri* AV19]
91	KOL01385	1254157	1254639	+	160	3-isopropylmalate dehydratase small subunit [*Methanopyrus kandleri*]
92	KOL01386	1254732	1255148	+	138	Uncharacterized protein conserved in archaea [*Methanopyrus kandleri* AV19]

93	KOL01387	1255206	1255847	+	213	hypothetical protein [*Methanobacterium congolense*]
94	KOL01388	1255837	1256160	+	107	hypothetical protein [*Methanobacterium congolense*]
95	KOL01389	1256176	1256709	+	177	hydrogenase 2 large subunit [*Corynebacterium glucuronolyticum*]
96	KOL01390	1256664	1258517	+	617	_
97	KOL01391	1258545	1259231	-	228	Uncharacterized, MobA-related protein [*Methanopyrus kandleri* AV19]
98	KOL01392	1259237	1260403	-	388	Predicted GTPase of the OBG/HflX superfamily [*Methanopyrus kandleri* AV19]
99	KOL01393	1260408	1261001	-	197	Integral membrane protein of the MarC family [*Methanopyrus kandleri* AV19]
100	KOL01394	1261065	1262714	+	549	RecName: Full=Dihydroxy-acid dehydratase; Short=DAD
101	KOL01395	1262711	1263310	+	199	Precorrin isomerase [*Methanopyrus kandleri* AV19]
102	KOL01396	1263291	1263776	-	161	Phosphoribosylcarboxyaminoimidazole (NCAIR) mutase [*Methanopyrus kandleri* AV19]
103	KOL01397	1263783	1264325	-	180	Uncharacterized membrane protein [*Methanopyrus kandleri* AV19]
104	KOL01398	1264330	1265583	-	417	RecName: Full=2,3-bisphosphoglycerate-independent phosphoglycerate mutase; Short=BPG-independent PGAM; Short=Ph
105	KOL01399	1265657	1265824	+	55	_
106	KOL01400	1265814	1266560	-	248	Uncharacterized conserved protein [*Methanopyrus kandleri* AV19]
107	KOL01401	1266557	1267102	-	181	Membrane-associated phospholipid phosphatase [*Methanopyrus kandleri* AV19]
108	KOL01402	1267256	1267543	+	95	Uncharacterized protein MK1190 [*Methanopyrus kandleri* AV19]
109	KOL01403	1267548	1269254	+	568	ATPase subunit of an ABC-type transport system, contains a duplicated ATPase domain [*Methanopyrus kandleri* AV19]
110	KOL01404	1269379	1269594	+	71	1,4-beta-xylanase [*Streptomyces* sp. JHA19]
111	KOL01405	1269622	1269756	+	44	_
112	KOL01406	1269853	1270830	-	325	Predicted extracellular polysaccharide hydrolase of the endo alpha-1,4 polygalactosaminidase family [*Methanopyrus kandleri* AV19]
113	KOL01407	1271253	1271489	-	78	_
114	KOL01408	1271921	1272139	+	72	_
115	KOL01409	1272295	1272432	+	45	_
116	KOL01410	1272892	1273254	+	120	Uncharacterized protein MK1185 [*Methanopyrus kandleri* AV19]
117	KOL01411	1273705	1274493	+	262	polyferredoxin [Methanopyrus polyferredoxin [*Methanopyrus kandleri* AV19]
118	KOL01412	1274641	1274946	+	101	hypothetical protein S40288_02547 [*Stachybotrys chartarum* IBT 40288]
119	KOL01413	1274988	1275395	+	135	Uncharacterized protein MK1179 [*Methanopyrus kandleri* AV19]
120	KOL01414	1275472	1275618	-	48	_
121	KOL01415	1275990	1276439	+	149	hypothetical protein S40288_02547 [*Stachybotrys chartarum* IBT 40288]
122	KOL01416	1276490	1276618	+	42	_
123	KOL01417	1276771	1277934	-	387	coenzyme F420-reducing hydrogenase, beta subunit [*Methanopyrus kandleri]*
124	KOL01418	1277947	1279584	-	545	Coenzyme F420-reducing hydrogenase, beta subunit [*Methanopyrus kandleri* AV19]

125	KOL01419	1279597	1279992	-	131	Selenocysteine-containing anaerobic dehydrogenase [*Methanopyrus kandleri* AV19]
126	KOL01420	1280682	1281074	+	130	Uncharacterized protein MK1175 [*Methanopyrus kandleri* AV19]
127	KOL01421	1281768	1282352	-	194	Predicted membrane protein [*Methanopyrus kandleri* AV19]
128	KOL01422	1282709	1282888	-	59	short-chain dehydrogenase [*Entamoeba dispar* SAW760]
129	KOL01423	1284330	1284509	+	59	_
130	KOL01424	1284544	1284855	+	103	_
131	KOL01425	1284861	1285121	-	86	_
132	KOL01426	1285284	1286153	+	289	Uncharacterized protein MK1169 [*Methanopyrus kandleri* AV19]
133	KOL01427	1286289	1286606	+	105	_
134	KOL01428	1286983	1287906	-	307	Uncharacterized secreted protein specific for *M.kandleri*, MK-6 family [*Methanopyrus kandleri* AV19]
135	KOL01429	1288308	1288745	+	145	Uncharacterized protein MK1611 [*Methanopyrus kandleri* AV19]
136	KOL01430	1288947	1290194	+	415	Uncharacterized conserved protein [*Methanopyrus kandleri* AV19]
137	KOL01431	1290635	1290766	+	43	_
138	KOL01432	1290800	1291516	+	238	Predicted nuclease of the micrococcal nuclease (thermonuclease) family [*Methanopyrus kandleri* AV19]
139	KOL01433	1291760	1293319	-	519	Uncharacterized domain specific for *M.kandleri*, MK-2 family [*Methanopyrus kandleri* AV19]
140	KOL01434	1293319	1293462	-	47	_
141	KOL01435	1294252	1294371	-	39	_
142	KOL01436	1294992	1295132	-	46	_
143	KOL01437	1295452	1295574	+	40	_
144	KOL01438	1295888	1296145	-	85	_
145	KOL01439	1296824	1297795	+	323	xylanase/chitin deacetylase [*Methanopyrus kandleri*]
146	KOL01440	1297832	1298224	+	130	_
147	KOL01441	1298264	1299310	-	348	Predicted membrane-bound metal-dependent hydrolase [*Methanopyrus kandleri* AV19]
148	KOL01442	1299316	1299852	-	178	Zn-dependent hydrolase [*Methanopyrus kandleri]*
149	KOL01443	1299892	1300122	-	76	Zn-dependent hydrolase [*Methanopyrus kandleri* AV19]
150	KOL01444	1300123	1301403	-	426	Uncharacterized protein specific for M.kandleri, MK-42 family [*Methanopyrus kandleri* AV19]
151	KOL01445	1301669	1302115	+	148	PREDICTED: X-linked retinitis pigmentosa GTPase regulator [*Manis javanica*]
152	KOL01446	1302117	1303316	+	399	Uncharacterized domain specific for *M.kandleri*, MK-2 family [*Methanopyrus kandleri* AV19]
153	KOL01447	1303459	1303812	+	117	_
154	KOL01448	1303809	1304108	+	99	_
155	KOL01449	1304322	1304492	-	56	_
156	KOL01450	1304517	1305215	-	232	_
157	KOL01451	1305270	1305440	-	56	_

158	KOL01452	1305453	1305596	-	47	_
159	KOL01453	1305945	1306064	-	39	_
160	KOL01454	1306138	1306626	-	162	Uncharacterized protein MK1338 [*Methanopyrus kandleri* AV19]
161	KOL01455	1306672	1306860	+	62	_
162	KOL01456	1307425	1307571	+	48	_
163	KOL01457	1307686	1308309	+	207	_
