# Supplementary material for: Comparative Genomics of Methanopyrus sp. SNP6 and KOL6 Revealing Genomic Regions of Plasticity Implicated in Extremely Thermophilic Profiles
Source: Front Microbiol. 2017 Jul 11;8:1278. doi: 10.3389/fmicb.2017.01278 (PMC5504354; doi:10.3389/fmicb.2017.01278)
Supplement: Supplementary file 4 [file Image1.PDF]

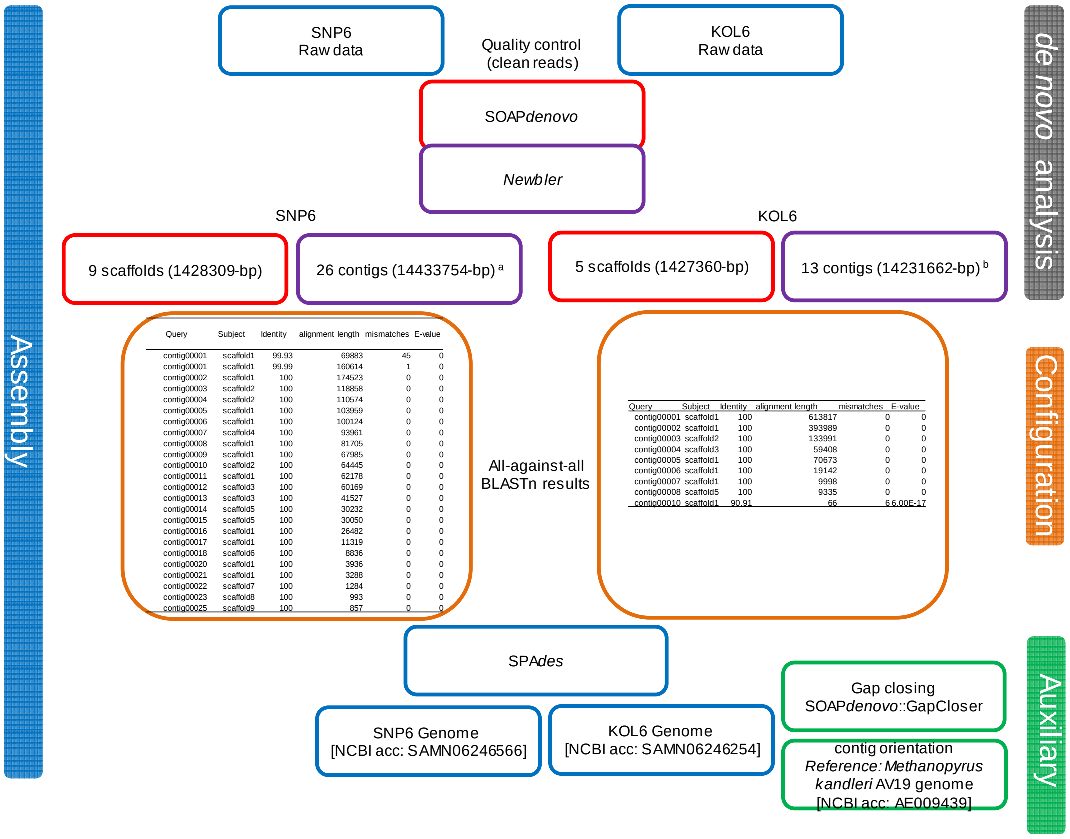

Query	Subject	Identity	alignment length	mismatches	E-value
contig00001	scaffold1	99.93	69883	45	0
contig00001	scaffold1	99.99	160614	1	0
contig00002	scaffold1	100	174523	0	0
contig00003	scaffold2	100	118858	0	0
contig00004	scaffold2	100	110574	0	0
contig00005	scaffold1	100	103959	0	0
contig00006	scaffold1	100	100124	0	0
contig00007	scaffold4	100	93961	0	0
contig00008	scaffold1	100	81705	0	0
contig00009	scaffold1	100	67985	0	0
contig00010	scaffold2	100	64445	0	0
contig00011	scaffold1	100	62178	0	0
contig00012	scaffold3	100	60169	0	0
contig00013	scaffold3	100	41527	0	0
contig00014	scaffold5	100	30232	0	0
contig00015	scaffold5	100	30050	0	0
contig00016	scaffold1	100	26482	0	0
contig00017	scaffold1	100	11319	0	0
contig00018	scaffold6	100	8836	0	0
contig00020	scaffold1	100	3936	0	0
contig00021	scaffold1	100	3288	0	0
contig00022	scaffold7	100	1284	0	0
contig00023	scaffold8	100	993	0	0
contig00025	scaffold9	100	857	0	0

Query	Subject	Identity	alignment length	mismatches	E-value
contig00001	scaffold1	100	613817	0	0
contig00002	scaffold1	100	393989	0	0
contig00003	scaffold2	100	133991	0	0
contig00004	scaffold3	100	59408	0	0
contig00005	scaffold1	100	70673	0	0
contig00006	scaffold1	100	19142	0	0
contig00007	scaffold1	100	9998	0	0
contig00008	scaffold5	100	9335	0	0
contig00010	scaffold1	90.91	66	6	6.00E-17

Figure S1. **KOL6 and SNP6 genome quality control and assembly strategies in this study.** Blue module denotes the essential procedures, whereas green the optional pipeline if applicable. SOAP*denovo* (red) and Newbler (purple) generated sequences of SNP6 and KOL6 have respectively been analyzed to examine the sequencing quality by using BLASTn-based all-against-all alignments (results marked in orange). Lastly, gap closing and contig orientation identification of SNP6 and KOL6 were consequently implemented via the SPA*des* software, SOAPdenovo::GapCloser program and available *Methanopyrus kandleri* AV19 genome as reference. [a]: Remove contigs 24 and 26 due to few number of detected reads, and non-homologs against SOAP*denovo* results found in contig 24 and 26. [b]: Remove contigs 9, 11, 12 and 13 due to few number of detected reads, and 1428994-bp left in 9 contigs.
