# Supplementary material for: Comparative Genomics of Methanopyrus sp. SNP6 and KOL6 Revealing Genomic Regions of Plasticity Implicated in Extremely Thermophilic Profiles
Source: Front Microbiol. 2017 Jul 11;8:1278. doi: 10.3389/fmicb.2017.01278 (PMC5504354; doi:10.3389/fmicb.2017.01278)
Supplement: Supplementary file 5 [file Image2.PDF]

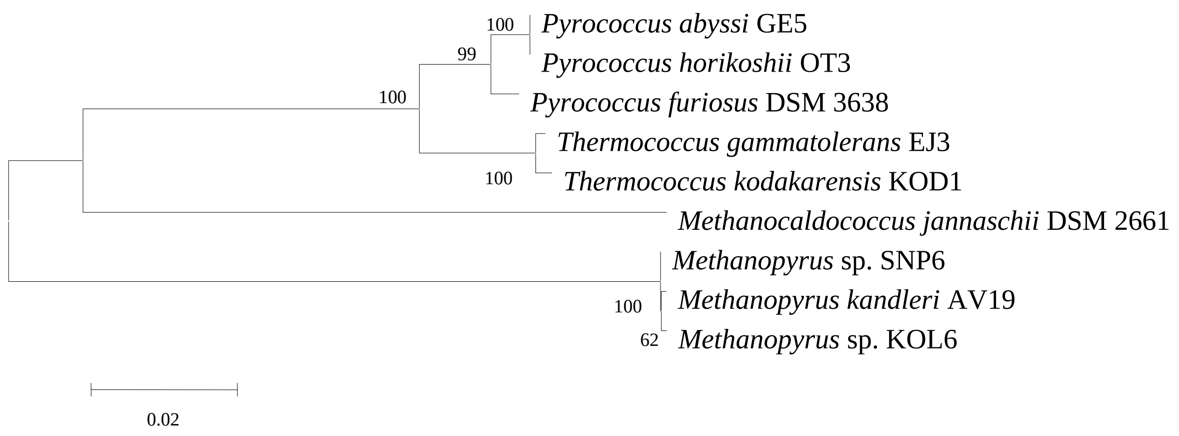

Figure S2. Phylogenetic analysis of *Methanopyrus* strains SNP6 and KOL6 based on the 16S rRNA gene sequences. Using the MEGA-embedded maximum likelihood approaches with bootstrap 1000 replicates. The phylogeny indicates that SNP6 and KOL6 both are closely-related species of strain AV19
