# Supplementary material for: Comparative Genomics of Methanopyrus sp. SNP6 and KOL6 Revealing Genomic Regions of Plasticity Implicated in Extremely Thermophilic Profiles
Source: Front Microbiol. 2017 Jul 11;8:1278. doi: 10.3389/fmicb.2017.01278 (PMC5504354; doi:10.3389/fmicb.2017.01278)
Supplement: Supplementary file 6 [file Image3.PDF]

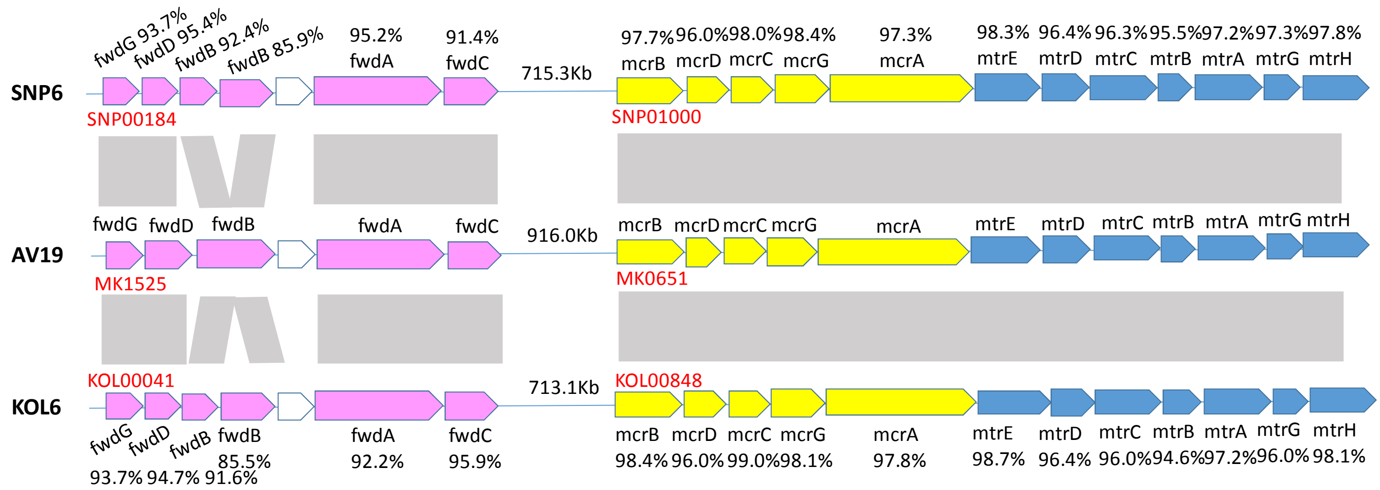

Figure S3. Gene organization and similarity (AV19 genes as subject) of the main methane metabolic pathway gene clusters in *Methanopyrus* strains SNP6 and KOL6, and *Methanopyrus kandleri* AV19.
