# Supplementary material for: Comparative Genomics of Methanopyrus sp. SNP6 and KOL6 Revealing Genomic Regions of Plasticity Implicated in Extremely Thermophilic Profiles
Source: Front Microbiol. 2017 Jul 11;8:1278. doi: 10.3389/fmicb.2017.01278 (PMC5504354; doi:10.3389/fmicb.2017.01278)
Supplement: Supplementary file 8 [file Image5.PDF]

A (SNP6 *vs.* AV19)

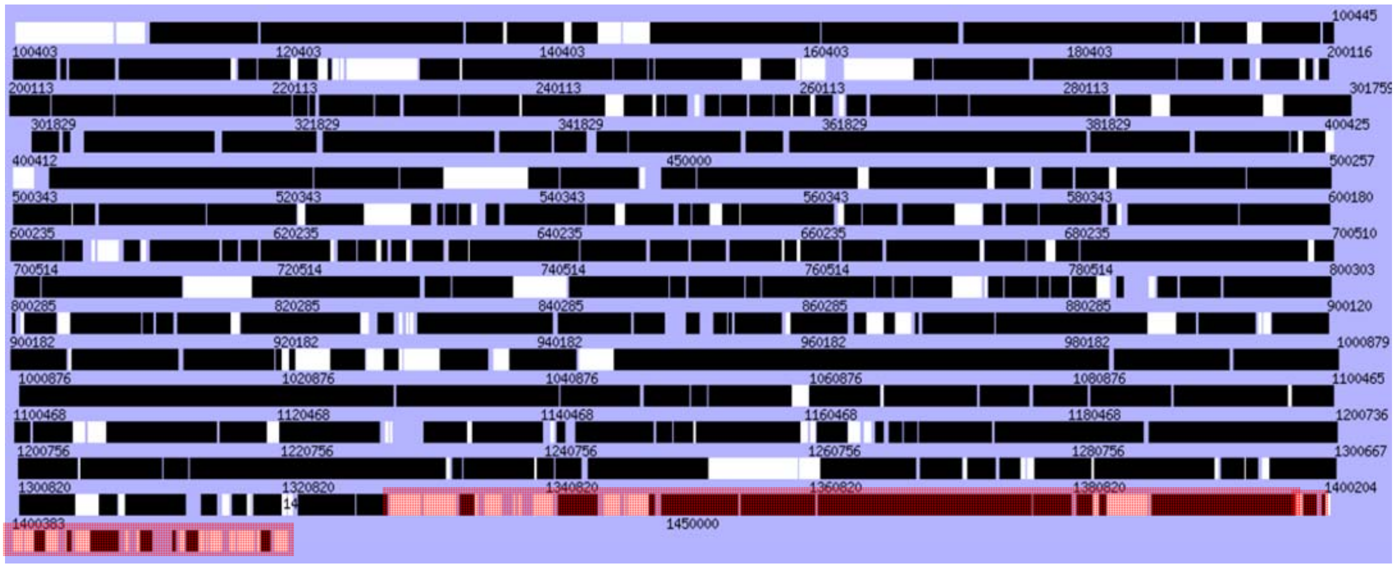

B (KOL6 *vs.* AV19)

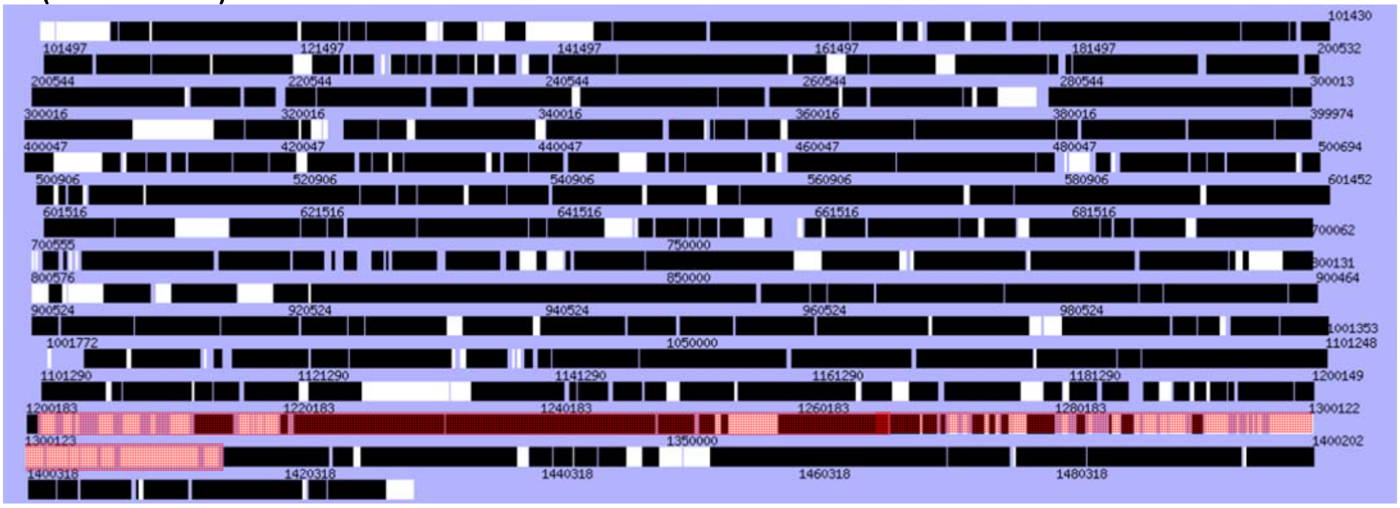

Figure S5. Identified large genomic regions of plasticity (RGP) in SNP6 (A) and KOL6 (B) against AV19. Genes shown in absolute black are conserved across all comparator genomes, while at the other extreme those shown in white are unique to AV19. Non-coding regions are shown as gaps, and each black-white linear rectangle was defined as 100-kb scale. Schematics of RGP and its homologs (red rectangle marked) in SNP6 and KOL6 are drawn to scale.
